# Molecular Characterization of an SV Capture Site in the Mid-Region of the Presynaptic CaV2.1 Calcium Channel C-Terminal

**DOI:** 10.3389/fncel.2018.00127

**Published:** 2018-05-11

**Authors:** Christine A. Snidal, Qi Li, Brittany B. Elliott, Henry K.-H. Mah, Robert H. C. Chen, Sabiha R. Gardezi, Elise F. Stanley

**Affiliations:** Presynaptic Mechanisms Laboratory, Krembil Research Institute, University Health Network, Toronto, ON, Canada

**Keywords:** presynaptic, calcium channel, synaptic vesicle tether, CaV2.1, P-type, channel C-terminal, synaptic vesicle binding domain, fast transmitter release

## Abstract

Neurotransmitter is released from presynaptic nerve terminals at fast-transmitting synapses by the action potential-gating of voltage dependent calcium channels (CaV), primarily of the CaV2.1 and CaV2.2 types. Entering Ca^2+^ diffuses to a nearby calcium sensor associated with a docked synaptic vesicle (SV) and initiates its fusion and discharge. Our previous findings that single CaVs can gate SV fusion argued for one or more tethers linking CaVs to docked SVs but the molecular nature of these tethers have not been established. We recently developed a cell-free, *in vitro* biochemical assay, termed SV pull-down (SV-PD), to test for SV binding proteins and used this to demonstrate that CaV2.2 or the distal third of its C-terminal can capture SVs. In subsequent reports we identified the binding site and characterized an SV binding motif. In this study, we set out to test if a similar SV-binding mechanism exists in the primary presynaptic channel type, CaV2.1. We cloned the chick variant of this channel and to our surprise found that it lacked the terminal third of the C-terminal, ruling out direct correlation with CaV2.2. We used SV-PD to identify an SV binding site in the distal half of the CaV2.1 C-terminal, a region that corresponds to the central third of the CaV2.2 C-terminal. Mutant fusion proteins combined with motif-blocking peptide strategies identified two domains that could account for SV binding; one in an alternatively spliced region (E44) and a second more distal site. Our findings provide a molecular basis for CaV2.1 SV binding that can account for recent evidence of C-terminal-dependent transmitter release modulation and that may contribute to SV tethering within the CaV2.1 single channel Ca^2+^ domain.

## Introduction

The finding that a single voltage-gated calcium channel (CaV) can gate the release of a synaptic vesicle (SV) at the presynaptic terminal of fast synapses was held to imply that the channel and the docked vesicle must be linked by a protein tether (Stanley, 1993, 1997). Accumulating evidence for the single channel gating model has greatly strengthened the argument in favor of such a link (Moser et al., 2006; Wang et al., 2009; Eggermann et al., 2011; Matveev et al., 2011; Hallermann and Silver, 2013; Meriney and Dittrich, 2013). A number of potential mechanisms of SV tethering have been proposed with molecular bridges that range from direct attachment to an SV integral protein to a variety of intermediary bridging proteins (Catterall, 1999; Hibino et al., 2002; Kiyonaka et al., 2007). We and others have hypothesized two primary presynaptic scaffolds: one that localizes the channel to the active zone and a second, reversible link that brings the SV within range of the single channel calcium domain (Harlow et al., 2001; Wong and Stanley, 2010) but the molecular identity of the latter SV tethering mechanism remains unresolved.

We recently developed a cell-free, *in vitro* assay that we term SV pull-down (SV-PD), to test directly for biochemical binding of SVs to biological and synthetic proteins and used this method to demonstrate that the SV can be captured by isolated, native CaV2.2 channels and specifically, by the channel C-terminal (Wong et al., 2013). Further studies using an array of C-terminal fusion proteins and blocking peptides identified an SV binding region just proximal to the C-terminal tip (Wong et al., 2014) and characterized an SV binding motif (Gardezi et al., 2016). Mimetic peptide competitive blockers of this motif loaded into chick synaptosomes inhibited SV turnover, as assessed by styryl dye recycling (Gardezi et al., 2016). Electron micrographic analysis of untethered-SV-vacated synaptosomes identified a small population of luminal SVs that were observed to be attached as far as ~200 nm from the surface membrane via faint fibrous processes (Wong et al., 2014). Further, nanogold immunolabeling suggests that these tethers include the unraveled channel C-terminal (Chen et al., 2017). Such long tethers could contribute to SV loading into the docking site near the channel but shorter links might be predicted to account for the short, ~25 nm distance, requirement for single domain gating (Stanley, 1993, 2015; Matveev et al., 2011; Dittrich and Meriney, 2016).

In this study, we set out with the primary objective of testing if CaV2.1 channels, which are the predominant presynaptic transmitter release-gating channels at most vertebrate fast-transmitting synapses, also exhibit C-terminal SV binding domains as we reported for CaV2.2. A report published very recently (Lübbert et al., 2017), after the experimental work was completed for this project, provided an exciting functional parallel to this work. Using the calyx of Held preparation, these authors found that while deletion of the distal third of the CaV2.1 channel had little effect on transmitter release, deletion of a mid-region greatly inhibited transmitter release. As detailed below, our findings are complementary to that study and together they provide a unique molecular insight into C-terminal function with respect to SV biology (see “Discussion” section).

**Figure 1:**
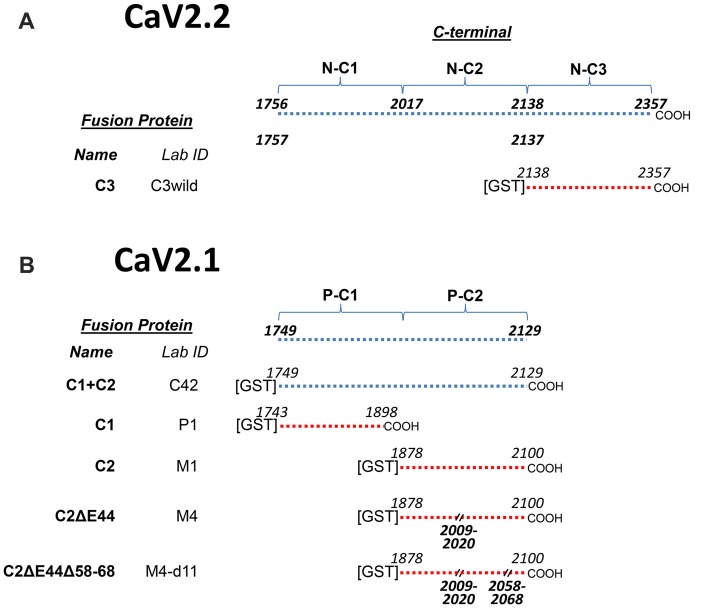
Constructs used in this project. **(A)** CaV2.2C-terminal constructs. The top line shows the full-length C-terminal and its operation division into three regions, C1, C2 and C3 together with the corresponding beginning and end amino acid sequence (aa) numbers. The CaV2.2 C3 region fusion protein, used as a control in this study, is detailed at the bottom with aa numbers. **(B)** CaV2.1C-terminal constructs. The full-length C-terminal diagram at the top lacks the distal third when compared to CaV2.2 **(A)** and is operationally divided into C1 and C2 regions to permit direct comparison to the CaV2.2 C-terminal (see text). Fusion proteins are listed below with the beginning and end aa numbers and aa deletions. Each fusion protein has two names, one unique identifier (Lab ID) and in bold text a functional name, as used in the text, reflecting its C-terminal third (C1, C2, C3 plus deletion codes).

At the onset of this project there was only a very small amino acid (aa) fragment of chick CaV2.1 in GenBank. Our work required a complete sequence for the C-terminal and hence, we first sequenced the chick CaV2.1 channel (NCBI Reference Sequence: KY353011). We found that the chick CaV2.1 was truncated at a point that is equivalent to exon 47 in humans (we refer to the human equivalent exons throughout). While short C-terminal-splice variants are common for both CaV2.1 and CaV2.2 in other species (Mori et al., 1991; Lü and Dunlap, 1999; Soong et al., 2002) the chick was unusual as numerous attempts to also detect the longer splice variant failed. Thus, while the C-terminal in CaV2.2 could be divided into three regions C1, C2 and C3, in essence CaV2.1 only exhibited C1 and C2 regions (Figures 1A,B). Obviously, this finding ruled out the possibility that SV-capture by the C-terminal C3 region, as demonstrated for CaV2.2, plays a significant role in SV recycling with CaV2.1. This finding provided the primary impetus for this study and we set out to test whether the truncated CaV2.1 C-terminal was indeed able to capture SVs and, if so, by what mechanism. We report a second C-terminal SV binding site within the C2 region of the chick CaV2.1 channel.

## Materials and Methods

### Molecular Procedures

#### CaV2.1 C-Terminal Fusion Protein Constructs

Chick CaV2.1 C-terminal constructs C1-C2 (residues 1748–2127), C1 (residues 1743–1898), and C2 (residues 1899–2100) were generated using RT-PCR. Gene-specific forward and reverse primers (GSPs) were designed using the chick CACNA1A (CaV2.1 alpha 1 subunit) C-terminal nucleotide sequence (Supplementary Table S1). The primers ranged from 17 to 41 nucleotides, contained at least five nucleotides that were different than the chick CACNA1B (CaV2.2 alpha 1 subunit) C-terminal sequence, and had a guanine-cytosine (GC) ratio of ~3:2. Each RT-PCR reaction consisted of 10 μL primer solution and 90 μL of double-distilled H_2_O.

#### CaV2.1 C-Terminal Construct RT-PCR

Fusion protein constructs encoding the CaV2.1 C-terminal regions were sub-cloned into the pGEX-KG vector as described (Chan et al., 2007). All constructs were transformed into *E. Coli* BL21 (DE3) cells (New England Biolabs, Ipswich, MA, USA), as previously described (Chan et al., 2007). In frame DNA sequence of each construct was confirmed by dideoxy sequencing (ACGT, Toronto, ON, Canada). All fusion protein constructs were transformed into DH5α competent cells (Invitrogen, Carlsbad, CA, USA) to confirm that the DNA sequence was in frame (ACGT, Toronto, ON, Canada).

#### CaV2.2 C-Terminal Region Fusion Protein Constructs

The CaV2.2 distal C terminal fusion protein, C3 (Figure 1A), was used as a positive control (see Wong et al., 2014; where it was termed C3_WildF_).

#### Fusion Protein Purification

Fusion proteins were purified using standard procedures (Chan et al., 2007; Gardezi et al., 2013). Briefly, proteins were expressed in BL21(DE3) cells and induced using isopropyl-β-D-thiogalactopyranoside (IPTG). Bacterial cell pellets were lysed using 1× PBS (Thermo Fisher Scientific, Waltham, WA, USA) supplemented with 1% Tween-20 (Bio-Rad), 0.01% Beta-mercaptoethanol, protease inhibitor cocktail (Sigma Aldrich), and 0.1 mM phenylmethanesulfonyl fluoride (PMSF) followed by sonication on ice using cycles of 10 s burst and 10 s rest. Cell lysate was centrifuged at 2400× *g* for 15 min. The supernatant was incubated with glutathione sepharose 4B beads (GE Healthcare, Little Chalfont, UK) for 2–3 h at 4°C. Immobilized fusion proteins were washed 3× with PBS supplemented with 0.05% Tween-20, 2× with PBS, 1× with HB (0.32 M sucrose, 2 mM EDTA, 10 mM HEPES; pH 7.4) and 2× with SV-PD buffer (0.32 M sucrose, 2 mM EDTA, 5 mM EGTA, 0.2857 mM CaCl2 (to give 10 nM Ca^2+^), 10 mM HEPES pH 7.4). Fusion proteins were used on bead for SV-PD assays. All the steps were carried out on ice and buffers were supplemented with protease inhibitor cocktail and 0.01 mM PMSF.

#### CaV2.1 Antibodies

Two new antibodies were generated for this study: PmidC2 and PC2var. PmidC2 antibody was generated against a LGTDLSVTTQSGDLPS peptide replicating a sequence in the middle region of the chick CaV2.1 C-terminal, while PC2var antibody was generated against a RRKVRPRGNNL peptide replicating the E44 alternative splice sequence (see “Results” section). These peptides were made with a terminal cysteine to permit conjugation to a carrier protein, KLH (Biomatik, Cambridge, Canada). Immunization of rabbits were done as previously described (Chen et al., 2017). All antibodies used in the present study are listed in Table 1.

**Table 1:** List of Antibodies used in this study.

Antibody name	Clonality	Target	Source	Dilution used for WB	Dilution used for ICC
Calbindin	Monoclonal (Clone D28k)	Calbindin	Synaptic Systems	-	1:200
PC2Var	Polyclonal	Chick CaV2.1 C-terminal (*alternatively spliced site, sequence RRKVRPRGNNL*)	Stanley lab	1:1000	1:200
PmidC2	Polyclonal	Chick CaV2.1 C-terminal (*middle region, sequence LGTDLSVTTQSGDLPS*)	Stanley lab	1:200	1:3000 to 1:1000
SV2	Monoclonal (Clone 17G10)	Synaptic vesicle glycoprotein 2A	Synaptic Systems	1:1000	-
SV2	Monoclonal (Clone SV2)	Synaptic vesicle glycoprotein 2A/B/C	Developmental Studies Hybridoma Bank	-	1:2
Synaptotagmin	Monoclonal (Clone ASV30)	Synaptotagmin-1	Abcam	1:1000	-
V-ATPase	Polyclonal	Vacuolar-type H^+^ ATPase	Santa Cruz Biotechnology	1:1000	-

### Biochemical Procedures

#### Synaptosome and Synaptic Vesicle Fractionation

The synaptosome and SV fractionation method has been described in detail (Gardezi et al., 2016). Briefly, E14-E17 chick brains (typically 105 brains per preparation) were homogenized. Synaptosomes were isolated by differential sucrose density gradient centrifugation and then ruptured by osmotic shock to release their contents. List of Antibodies used in this study. SVs were then isolated and purified by differential sucrose density gradient centrifugation. The SVs were maintained intact in detergent-free buffer for all experiments. Key buffers were: homogenization buffer (HB), 0.32 M sucrose, 10 mM HEPES, 2 mM EDTA, pH 7.4; and osmotic rupture buffer, 50 mM HEPES, 2 mM EDTA, pH 7.4 (Wong et al., 2014; Gardezi et al., 2016).

#### Western Blot

Standard Western blotting (WB) method was carried out as described previously (Wong et al., 2013, 2014) and immunoblots were imaged using a ChemiDoc™ XRS System (Bio-Rad, Hercules, CA, USA) as described (Gardezi et al., 2016).

#### Fusion Protein Concentrations and Blot Figures

It was essential to ensure that the concentrations of fusion protein on the beads were comparable. As a first step, for each fusion protein we added a range of concentrations to the precipitation beads and used Coomassie staining to identify a concentration that did not saturate the gel and was reasonably comparable to that of our other proteins (*data not shown*). In initial SV-PD experiments we fine-tuned the method by running several (2–4) concentrations of the new fusion protein. SV-PD analysis was only carried out on lanes where the Coomassie fusion protein bands were comparable in intensity. Thus, each experiment had up to 12 actual SV-PD trials (for example see Supplementary Figure S1) but only the lanes with comparable fusion protein concentration were used for analysis. The blot was imaged at a range of exposure durations and the duration that had the darkest bands without saturation of the SV-PD lanes was selected for further analysis. Any contrast adjustments for final figures were applied to the entire blot. The last step was to crop the gel image to present only the lanes that were relevant to the experiment in question and that contributed to the quantitative analysis. Hence, the immunoblots in each figure are all from the same experiment and care was taken to ensure that their relative intensities were maintained for display.

#### Synaptic Vesicle Pull-Down Assay

The SV pull-down method (SV-PD) has been described in detail (Wong et al., 2013, 2014). Briefly, purified SVs were incubated with immobilized fusion protein or control GST in a detergent free, SV-PD buffer (HB with 5 mM EGTA and (Ca^2+^) clamped to 10 nM by addition of CaCl_2_ as calculated with MaxChelator, maxchelator.stanford.edu). Forty microliter of the SV suspension, containing the SV sample used for pull-down assays, was reserved for WBs. SV-PD samples were washed four times with SV-PD buffer and solubilized in 2× Laemmli sample buffer (Bio-Rad) with 5% β-mercaptoethanol. Samples were boiled for 5 min at 100°C, cooled on ice and then run on 8%–12% discontinuous polyacrylamide gels for WBs. Immunoblots were probed for integral SV membrane proteins as markers for vesicle capture. SV-PD was considered positive if the band intensity of at least two SV integral membrane marker proteins, generally SV2, STG or v-ATPase, were more intense than for control samples. Our decision to only use freshly-prepared SVs (to prevent storage artifacts) made these experiments time-demanding and limited us to typically one experiment per week. It should also be noted that detergent-free biochemical methods, using intact lipid-bound structures are prone to higher levels of non-specific SV capture, dictating repeated trials and statistical analysis to form a conclusion. For unknown reasons GST-probed blots were particularly variable with respect to non-specific binding and could not be used for quantification. Criteria for acceptance for further analysis were based on a rejection of blots with a significant level of non-specific binding (“Materials and Methods” section described in Wong et al., 2014; Gardezi et al., 2016).

#### Immunoblot Quantification and Analysis

Immunoblots were analyzed as described in Gardezi et al. (2016). Immunoblots were imaged with the ChemiDoc (Bio-Rad, Hercules, CA, USA) with a broad range of exposure times. For each experiment, protein band intensities were quantified by densitometry from a common blot at a single exposure selected for clear bands without saturation. Background counts were subtracted using an automated rolling disk subtraction. Protein band intensities were normalized to a single control condition for each experiment. For experiments comparing different fusion proteins, intensities were normalized to the C2 fusion protein intensity. For peptide experiments, intensities were normalize to the control peptide condition as described previously (Gardezi et al., 2016).

Fusion protein concentrations were visualized using Coomassie stain (Sigma-Aldrich) and imaged with the ChemiDoc (Bio-Rad) Coomassie stain protein gel function. Concentrations were quantified by densitometry and background was subtracted using an automated rolling disk subtraction. Fusion protein concentration was used as a loading control so that SV2 (I_SV2_) and STG (I_STG_) protein intensities were normalized to the fusion protein concentration (I_FP_) from the same lane, hence %SV-PD was calculated as I_SV2_/I_FP_ or I_STG_/I_FP_ respectively.

#### CaV C-Terminal Region Mimetic Peptides

Control and putative blocking peptides were synthesized by Biomatik. SV binding-motif blocking and control peptides have been described previously (Gardezi et al., 2016). In the present study, HQARRAPNGA was used as the motif peptide and HQAGGAGGGA was used as a control peptide. Peptides were reconstituted in SV-PD buffer at 10 mM and added to SVs to obtain a final concentration of 0.6 mM. SVs were incubated with the peptides for 2–3 h at 4°C prior to pull-down with the fusion proteins.

### Statistical Analysis

Data are presented as mean ± standard error (SE) and with the number of independent experiments (each experiment started with a fresh brain homogenate) denoted by N. Statistical analysis was performed with GraphPad (San Diego, CA, USA) Prism 6.0. Mutant fusion protein SV-PD was tested using a one-sample, two-tailed *t*-test based on the null hypothesis that mean SV-PD = 100% of the result with the C2 fusion protein. The two mutant fusion proteins were tested against each other using a two-tailed, paired *t*-test. Each peptide treatment was tested using a one sample, two-tailed *t*-test based on the null hypothesis that mean SV-PD = 100% of the result with the control peptide, as described (Wong et al., 2014; Gardezi et al., 2016). Values were considered significantly different if *p* < 0.05.

## Results

### Calcium Channel C-Terminal Fusion Protein Constructs

#### CaV2.2 Fusion Proteins and Terminology

To facilitate experimental analysis, we divided the CaV2.2 C-terminal into three segments: C1, C2 and C3 (Figure 1A) and created corresponding normal and mutant fusion proteins (Wong et al., 2013, 2014; Gardezi et al., 2016). One of these, C3, was used for comparison with CaV2.1 fusion proteins in this study (Figure 1B). To simplify discussion, we renamed these fusion proteins according to their C-terminal region (Figure 1B). However, fusion protein laboratory names are also listed for cross-reference.

#### Chick CaV2.1 Cloning

At the onset of this project only a very short segment of the chick CACNA1A had been sequenced (NCBI Reference Sequence: XM_004950371.1). The library did, however, contain a fairly extensive but incomplete, “predicted” and hence, unconfirmed sequence for a distantly related bird, the ground pecker (*Pseudopodoces humilis*; NCBI Reference Sequence: XM_005533612.1). As neither library sequence included the C-terminal region, we cloned the full-length channel (Supplementary Figure S2; NCBI Reference Sequence: KY353011).

The chick CaV2.1 has a high homology with crocodile (the closest phylogenic preceding species) and the incomplete predicted ground pecker (Avian) sequence with, as expected, a lower homology to mammals (*data not shown*). However, we found that the C-terminal was truncated, lacking virtually the entire C3 region (Figure 1B). Attempts to identify a splice variant with a longer C-terminal were unsuccessful and we presume that the predominant chick CaV2.1 terminates at the HCMNRNN sequence (aa 2127). Thus, it appears that the chick CaV2.1 channel lacks our previously reported SV binding site just proximal to the C-terminal tip (see “Discussion” section). To facilitate study of our fusion proteins we raised an antibody, PmidC2, against a peptide (LGTDLSVTTQSGDLPS; aa 2068–2083) within the CaV2.1 C-terminal C2 region. This antibody was characterized by Western blot (WB) and immunocytochemistry (Supplementary Figures S3Bi,ii,iii). It identified our CaV2.1 C2, but not CaV2.1 C1 or CaV2.2 C2 region fusion proteins (Supplementary Figure S3Aii) and a band corresponding approximately to a CaV2 channel weight by WB (note, however, that molecular weights vary markedly between blots for these very large proteins; Supplementary Figure S3Ai). Immunocytochemistry demonstrated staining of the large chick cerebellar Purkinje somata and dendrites, the cells in which the channel was first identified (Llinás et al., 1989; Supplementary Figure S3Aiii).

**Figure 2:**
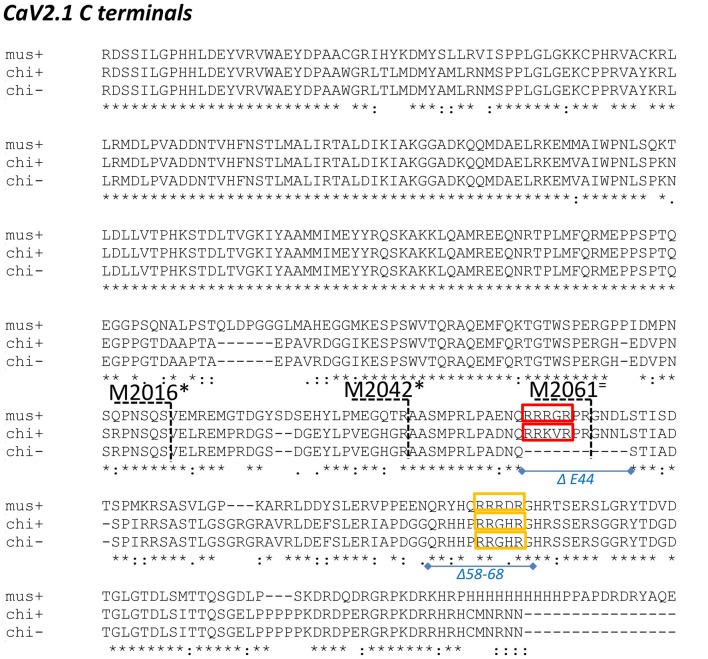
Alignment of chick and mouse CaV2.1 C termini. Chick is shown both with (+) and without (-) the E44 splice insert and the distal third of the mouse C-terminal is not shown. mus: mouse; chi+: full length chicken; chi-: chicken ΔE44 splice variant. Red and yellow boxes: candidate synaptic vesicle (SV) binding motifs in both species; blue lines and italic text: chick C-terminal deletions; black numbers/dashed lines: mouse C-terminal truncation points as in Lübbert et al. (2017). Truncations that inhibited release are marked with an * and those that did not with an = (see “Discussion” section).

By alignment with CaV2.2 we divided the CaV2.1 C-terminal into C1 and C2 regions, with the C2 region containing the terminus (Figure 1). During the cloning, we discovered a splice variant that lacked a short segment within the C2 region: aa sequence RRKVRPRGNNLS (aa 2009–2020; Figure 2). This splice variant has been described previously in mammalian CaV2.1 channels and corresponds to omission of exon 44 (Zhuchenko et al., 1997). We refer to fusion proteins that contain these two forms as the “C2” and “C2ΔE44” variants, respectively.

To analyze SV binding to the CaV2.1 C-terminal we generated five fusion proteins, including the full-length terminal (C1+C2), the C1 region (C1), C2 region (C2) and C2 without the splice (C2ΔE44) variants (Figure 1B).

### Synaptic Vesicles Capture By the Channel C-Terminal

#### SVs Are Captured By CaV2.1 C-Terminal

**Figure 3:**
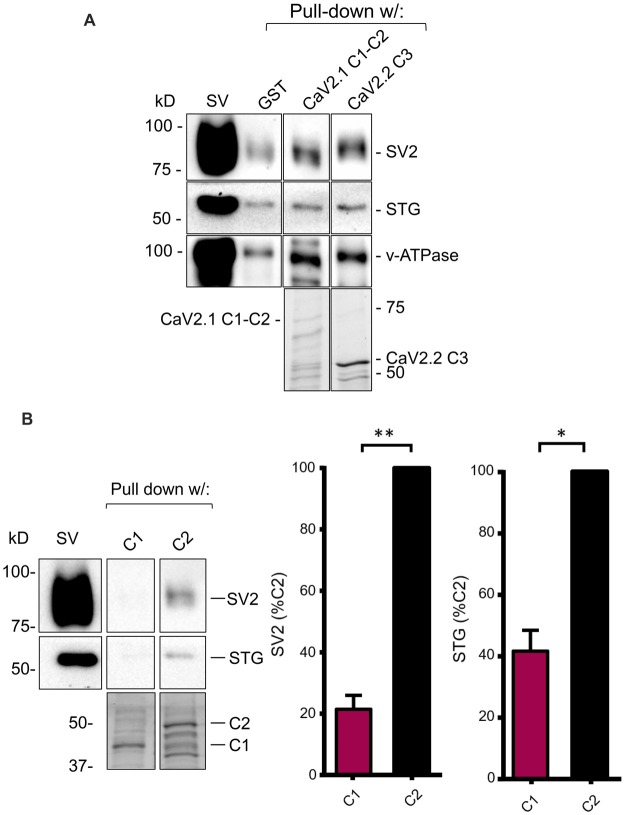
The C2 region of CaV2.1 binds SVs. **(A)** SV pull-down (SV-PD) using the full-length chick CaV2.1 C-terminal (C1+C2) assessed by SV2, STG and v-ATPase immunoblot. A positive control of SV-PD using the CaV2.2 channel C3 segment is shown for comparison. SV lane: western blot of the SV sample input. In this and all subsequent representative figures all the lanes were taken from a single blot from the same experiment at one given exposure time (see “Materials and Methods” section). **(B)** *Left panel*: as in **(A)** but showing SV-PD with C1 or C2 region fusion proteins. *Right panel*: histograms comparing SV and STG protein band intensities for the two fusion proteins. *p < 0.05, **p < 0.01. Fusion proteins were detected by Coomassie stain unless stated.

In our earlier reports, we demonstrated and characterized a SV binding site on the C3 segment of CaV2.2 (Wong et al., 2014; Gardezi et al., 2016). Since our cloned CaV2.1 channel has a truncated C-terminal lacking this region, we initially hypothesized that it would fail to also capture SVs. To test this, we carried out SV-PD using a fusion protein that comprised the entire CaV2.1 C-terminal, C1+C2 (Figure 1B). The finding of robust SV capture (*N* = 4; Figure 3A) contradicted this hypothesis and demonstrated that there had to be a novel SV binding site on the truncated CaV2.1 C-terminal.

#### SV Capture By C1-C2 Is Attributable to the C2 Region

To localize the SV binding site, we next created separate fusion proteins of the C1 and C2 regions (Figure 1B) and tested these for SV-PD. Consistently, the C1 region failed to capture SVs compared to strong capture by the C2 region (Figure 3B; C1 expressed as a percentage of C2: SV2 = 21.5 ± 4.5% *N* = 3, *p* < 0.01; STG = 41.6 ± 6.7% *N* = 3, *p* < 0.05). Thus, the SV capture observed with the C1+C2 fusion protein could be attributed to the C2 region.

#### A C2 Region SV Binding Site?

**Figure 4:**
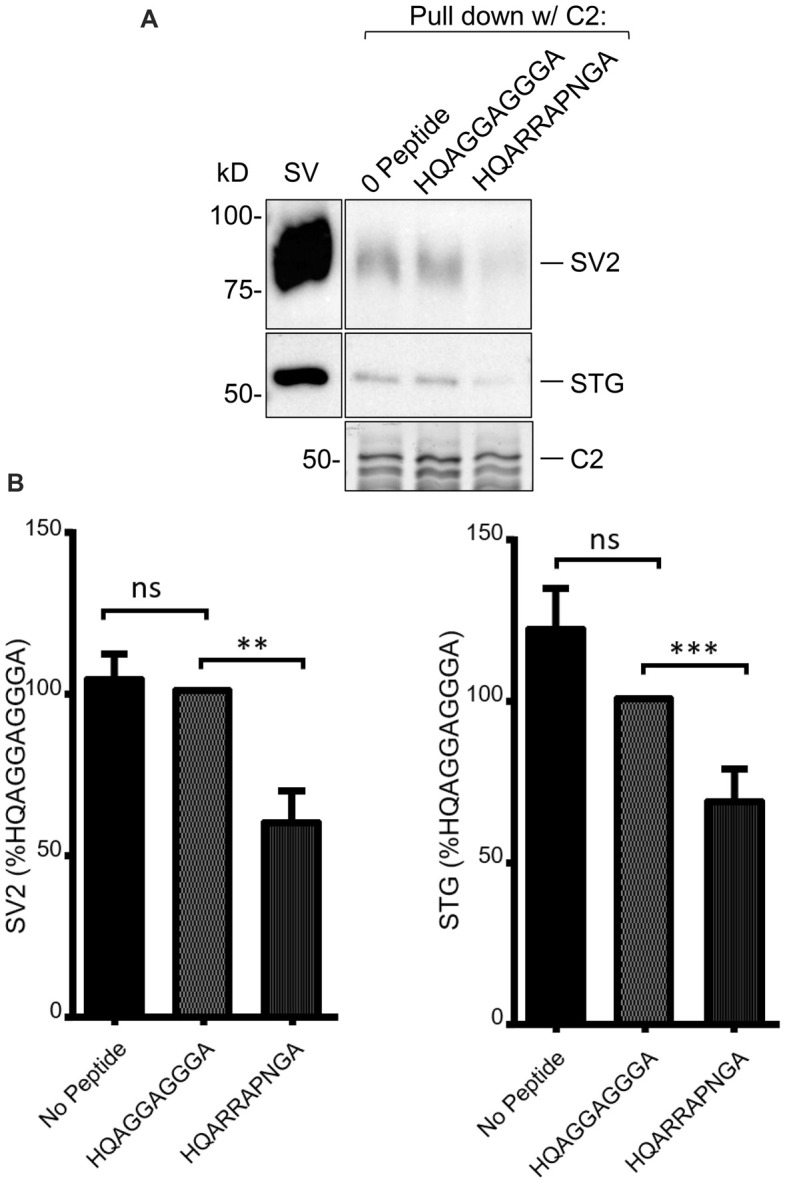
**(A)** SV-PD with C2 is inhibited by (+)xxRR motif mimetic blocking peptide. **(B)** *Left panel*: SV-PD with C2 fusion protein as in Figure 4A after no treatment (0 peptide) or pretreatment of the SV sample with control, HQAGGAGGGA, or motif-blocking peptide, HQARRAPNGA. Note the marked inhibition of SV protein capture with the blocking peptide. **(B)** *Right panel*: histograms comparing SV or STG protein band intensity for no peptide, control peptide and blocking peptide conditions. **p < 0.01; ***p < 0.001; ns *p* > 0.1.

As discussed above, we previously used mimetic blocking peptides to identify an SV binding motif, +(+)x RR (where + denotes a positively charged and x an unspecified residue, respectively) in the distal C3 region of the C-terminal (Gardezi et al., 2016). To test if SV capture in the C2 region involved the same motif we carried out SV-PD in the presence of a previously confirmed blocking peptides, HQARRAPNGA, or an inert control, HQAGGAGGGA (see Gardezi et al., 2016). Interestingly, pre-incubation of the SVs with the test peptide markedly reduced C2 fusion protein SV-PD compared to the control (Figure 4; C2+ blocking peptide SV-PD expressed as a percentage of the control peptide: SV2 = 59.8 ± 9.8% *N* = 14, *p* < 0.01; STG = 68.0 ± 10.3% *N* = 14, *p* = 0.01). Based on this finding we concluded that SV capture involved the same SV binding motif in the C2 region of CaV2.1 channels as previously characterized for the CaV2.2 C3 region.

#### The CaV2.1 SV Binding Site

**Figure 5:**
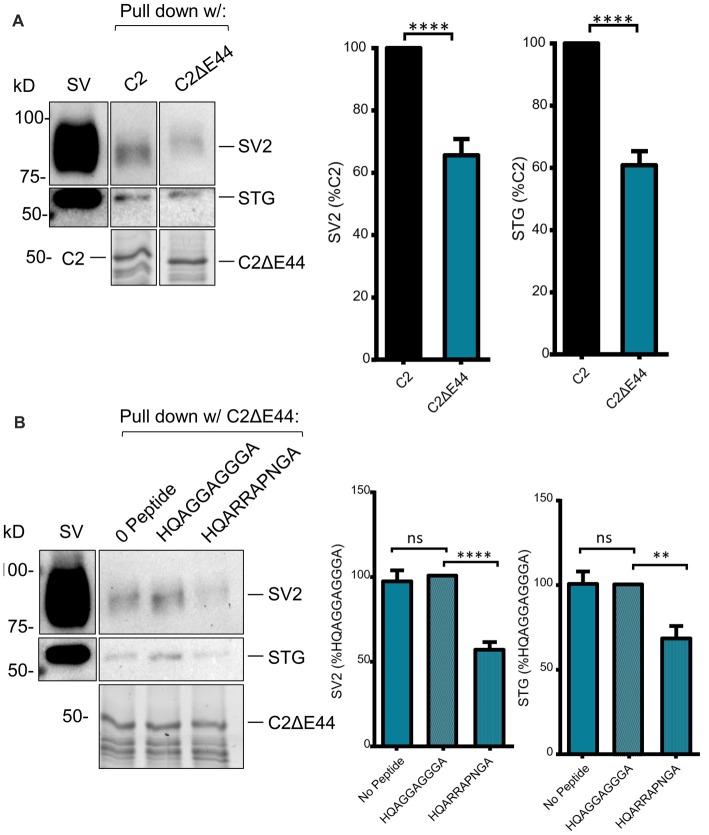
SV-PD with the C2ΔE44 mutant C2 fusion protein. **(A)** *Left panel*: SV-PD with the C2ΔE44 fusion proteins. *Right panel*: histograms comparing SV or STG protein band intensity for the two fusion proteins. ****p < 0.0001. **(B)** *Left panel*: The (+)xxRR motif blocking peptide HQARRAPNGA reduced but did not eliminate SV-PD using the C2ΔE44 fusion protein. *Right panel*: histogram comparing SV2 or STG protein band intensity for no peptide, control peptide and blocking peptide conditions. **p < 0.01; ***p < 0.001; ns *p* > 0.1.

We next scrutinized the amino acid sequences of the C2 region fusion protein to search for putative SV-binding motifs (Figure 2). As we have no evidence that these motifs are within structurally restricted regions (Gardezi et al., 2016) we included both ortho- (N-to-C) and antidromic (C-to-N) matches and identified one high-homology site: RRKVRPRGNNL (aa 2009–2020) plus one additional site (aa2059–2067) that exhibited motif-like sequences in both orthodromic, RHHPRRGHR, and antidromic, RHHPRRGHR directions. We were fascinated by the realization that the first of these sites, lay within the alternatively spliced E44 region and, hence, is present in the C2 but not the C2ΔE44 fusion proteins. Thus, we could evaluate SV binding to this site by comparing fusion proteins of the two natural C2 splice variants. We observed markedly weaker SV capture using C2ΔE44 compared to the C2 fusion protein (Figure 5A; C2ΔE44 SV-PD as a percentage of C2: SV2 = 65.6 ± 5.2 *N* = 25, *p* < 0.0001; STG = 61.7 ± 4.5 *N* = 23, *p* < 0.0001). Our data indicates, therefore, that a SV binding site is located within the alternatively spliced region of the CaV2.1 channel C-terminal. However, we also noted that although SV-PD was reduced with C2ΔE44, the blots suggested that it was not eliminated. To explore this possibility, we tested if SV protein capture using C2ΔE44 could be inhibited using the mimetic motif-blocking peptide, as above. This was the case (Figure 5B; C2ΔE44 with blocking peptide SV-PD as a percentage C2ΔE44 with control peptide: SV2 = 56.5 ± 4.6% *N* = 11, *p* < 0.0001; STG: = 68.1 ± 7.2% *N* = 11, *p* < 0.001), favoring involvement of the second, +(+)x RR, motif binding site within the C2 region.

**Figure 6:**
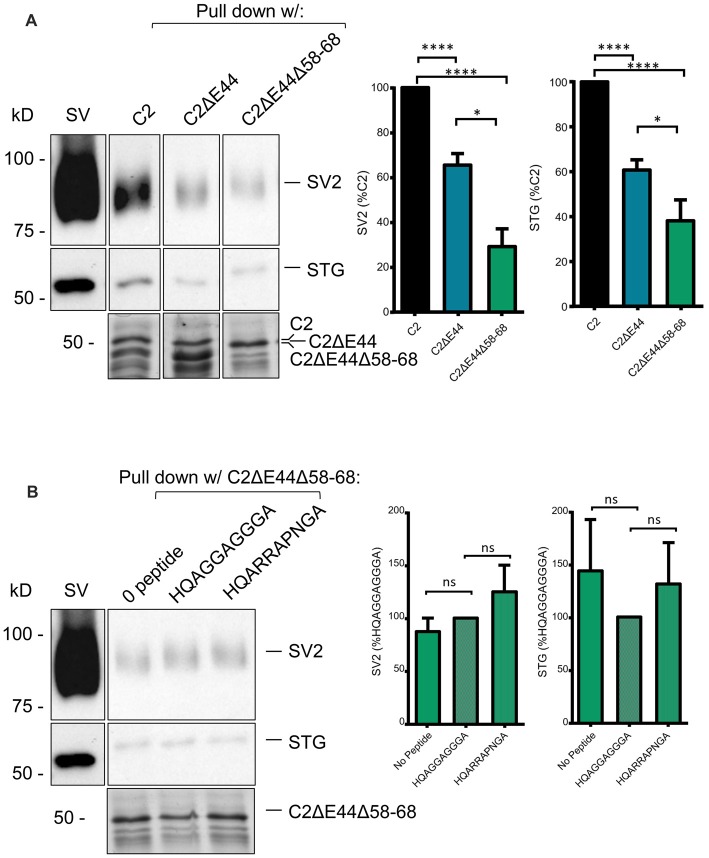
SV-PD with the double C2ΔE44Δ58–68 mutant C2 fusion protein. **(A)**. *Left panel*: SV-PD with the double-deletion C22ΔE44, Δ58–68 fusion protein. *Right panel*: histograms comparing SV2 or STG protein band intensity for the two fusion proteins. *p < 0.05, ****p < 0.0001. **(B)** Lack of effect of the blocking peptide on SV-PD with the double-deletion C22ΔE44Δ58–68 fusion protein. *Right panel*: histogram comparing SV protein band intensity for no peptide, control peptide and blocking peptide conditions. ns *p* > 0.1.

To test if the other SV binding motif could account for the residual SV-PD we created a mutant fusion protein that also lacked aa 2058–2068, C2ΔE44 Δ58–68 (Figure 1B). The deleted sequence, RHHPRRGHR, contains the antidromic SV-binding motif RRGHR as well as a possible (but perhaps unlikely due to the proline) orthodromic motif of HHPRR. SV protein capture using this fusion protein was reduced even further, as compared to the C2ΔE44 fusion protein. While this finding was only significant for SV2, we could detect a trend towards significant with STG as well (Figure 6A, C2ΔE44Δ58–68 SV-PD as a percentage of C2: SV2 = 28.7 ± 7.0%, *N* = 10; STG = 38.5 ± 8.5%, *N* = 11, *p* < 0.0001, N = 11, C2ΔE44 compared to C2ΔE44Δ58–68 SV2: *p* < 0.05; STG: *p* < 0.1). The double mutant effectively eliminated motif-dependent SV capture as the blocking peptide was now without effect (Figure 6B; C2ΔE44Δ58–68 with blocking peptide as a percentage of C2ΔE44Δ58–68 with control peptide: SV2 = 125.3 ± 25.4%, *p* > 0.1, *N* = 7; STG = 131.9 ± 39.4%, *p* > 0.1, *N* = 7).

## Discussion

The main findings of this study are that: the mid, C2 region of the chick CaV2.1 C-terminus can bind SVs; this vesicle capture is blocked by our previously characterized SV-binding site-motif mimetic peptide; mutation of identified putative binding motifs greatly reduces SV-PD, and a channel C-terminal splice-variant exhibits markedly reduced SV binding. Thus, we have compelling evidence for an SV binding site in the mid region of the CaV2.1 channel C terminus.

This study sprang from our original objective: to characterize SV binding to the C-terminal C3 region of the CaV2.1 channel. As there was very little information published on the molecular structure of chick CaV2.1 and nothing on its C-terminal we cloned the full-length channel. We were, however, unable to find a variant that exhibited the full-length C-terminal and, although it remains possible that a longer variant was missed, we had to conclude that in the chick this channel is truncated at point corresponding to the commencement of human Exon 47. Such a variant has been reported previously in humans and other mammals (Mori et al., 1991; Zhuchenko et al., 1997; Tsunemi et al., 2002). Thus, in comparison with CaV2.2, the chick CaV2.1 C-terminal comprised only the C1 and C2 regions, precluding a C3 region SV binding site. Thus, we set out to search for an alternative SV attachment site on the CaV2.1 C-terminal. We also noted a splice variant omitting a short exon that corresponds to Exon 44 in humans (Zhuchenko et al., 1997), referred to here as C2ΔE44.

We first generated a full-length CaV2.1 C-terminal fusion protein (Figure 1B, equivalent to a combination of the C1-C2 region of CaV2.2; Figure 1A). This fusion protein exhibited strong SV-PD and provided evidence in support of a novel SV binding site. We next created separate fusion proteins of the C1 and C2 regions (Figure 1B) and observed that SV-PD was only robust with C2 (Figure 3B). To test whether the C2 region SV capture involved the same motif as reported earlier, we tested if SV-PD could be inhibited by a previously characterized motif-mimetic peptide (Gardezi et al., 2016). A marked inhibition of SV-PD with this peptide argued that SVs were binding to the fusion protein via the same SV attachment mechanism as characterized for the C3 region of CaV2.2.

Scrutiny of the CaV2.1 C-terminal aa sequence identified an obvious (because of the strong aa positive charge) antidromic motif within the alternatively spliced, E44, aa sequence, RRKVRPRGNNL (Figure 2, red box). Thus, C2 region fusion proteins with or without the alternatively spliced region could be used as natural tests for SV capture. This binding site was supported by the finding that C2ΔE44 consistently exhibited weaker SV-PD than C2. However, some SV-PD persisted. We therefore re-examined the C2 sequence for additional putative SV binding motifs and identified a site with a complex ortho-/antidromic, putative motif RHHPRRGHR/RHHPRRGHR, respectively (Figure 2, yellow box). This site accounted for the residual SV-PD because a mutant fusion protein that lacked both motifs, C2ΔE44Δ58–68, failed to exhibit SV-PD (Figure 6A). To confirm this conclusion, we demonstrated that while the motif-blocking peptide reduced SV capture for both the C2 and C2ΔE44 fusion proteins (Figures 4, 5B), it was without effect on SV capture by the C2ΔE44Δ58–68 double deletion (Figure 6B). Thus, we can reasonably conclude that SV capture can be attributed to the two SV binding motifs and that these sites exhibit some degree of redundancy. It should be noted in an earlier study where the intent was to evaluate the functional role of the CaV2.2 C3 SV-binding site, we introduced motif-blocking peptides into freshly isolated synaptosomes and used styryl dye uptake to test for a transmitter release inhibition (Gardezi et al., 2016). We observed prominent inhibition and suggested that this argued for an important role for the CaV2.2 C3 site. We interpreted the strong block as an indication that CaV2.1 channels utilize a similar C3 binding site. The findings here suggest an alternative rationale: that the observed transmission inhibition reflects block of C2 region SV binding sites.

The recent publication of the report by Lübbert et al. (2017) provided us with a serendipitous functional test of our biochemical and molecular analysis. It should be stressed from the outset that while we were aware of the Young laboratory’s work in this area, including the finding that ablation of a part of the C-terminal inhibits transmitter release, until the article was published we were blinded to the molecular details. Lübbert et al. (2017) deleted the native CaV2.1 channel in mice and then replaced it with mutant channels with progressively shorter C termini. They then tested these for effects on the presynaptic CaV2.1 current and transmitter release at the calyx of Held synapse. In their experiments, they carried out a series of progressively shorter C-terminal “crops” and tested if these would affect the strength of transmitter release. Their mouse C-terminal crops are shown in Figure 2 together with the aligned chick C-terminal from this study and the locations of our identified SV binding sites. Lübbert et al. (2017) found that C-terminal pruning from 2365 or 2213 (both distal to the region shown in Figure 2) had no significant effect transmitter release, which is consistent with identified binding sites. In contrast, M2016 or the slightly more distal M2042, which remove both of our SV binding sites, markedly inhibited transmitter release, again consistent with our findings.

The intermediary, M2061, cut site in the Lübbert et al.’s (2017) study is of particular interest. This is located within the alternatively spliced, E44, region (Figure 2) and while pruning at this site effectively removes our Δ58–68 SV binding site, it leaves the more proximal RRRGR motif intact. This channel mutant did not cause a significant inhibition of release. Since there is a partial redundancy between the two binding sites (SV-PD persists when one site is deleted), the Lübbert et al. (2017) functional data is therefore remarkably consistent with our identified SV binding sites. Perhaps the only detail that our findings cannot explain is why the M2016 crop appears to be a bit more effective than M2042. This implies the existence of an additional release modulator within the region just proximal to our SV binding sites. Nonetheless, the similarity of the Lübbert et al. (2017) physiological data with our biochemical analysis is mutually supportive. Our findings provide a molecular basis for their C-terminal cropping data while theirs provides functional support not only for our identified SV binding sites but also for the general SV-PD analysis strategy.

Our results demonstrate SV capture by the CaV2.1 C2 region can be attributed to redundant RR-containing domains. We have not yet identified the binding partner of these domains on the SV. The simplest hypothesis is that this is located on a key intrinsic SV protein but an SV-associated protein is also a possibility. RIM1/2, an SV-associated protein, has been hypothesized to link SVs to the CaV (Hibino et al., 2002; Kiyonaka et al., 2007). However, while our previous results support the idea that the channel and RIM1 covary at the release site (Khanna et al., 2006) we have repeatedly failed to either confirm biochemical evidence for a stable molecular binding interaction nor evidence that RIM1/2 plays a role in SV binding to the C3 region of the CaV2.2 C-terminal, as assessed by our *in vitro* SV-PD assay (Wong et al., 2013, 2014; Gardezi et al., 2016). Since the same motif is at play here it is highly unlikely that RIM1/2 serves as an SV link in the CaV2.1 C2 region.

We have predicted that the SV-calcium sensor should be located within ~25 nm of the calcium channel pore to permit single domain-based SV gating (Stanley, 1993, 1997, 2016). Software predictions suggest that the C-terminal exhibits very little predicted secondary structure and its tip could, at least in theory, range far into the surrounding cytoplasm (Wong et al., 2014). We recently used nanogold immunocytochemistry to localize the different regions of the CaV2.2 C-terminal within cytoplasm-vacated, but tethered SV-retaining, nerve terminals (Chen et al., 2017). We found that the tip of the C-terminal, in the region corresponding to our first identified (C3) SV-binding motif, contacts SVs up to and over 100 nm from the active zone, at least in nerve terminals vacated of cytoplasmic constituents. However, an antibody against the C2 region of the C-terminal was associated with nanogold particles localized much closer to the active zone. The finding that these gold particles were also in contact with tethered SVs, argued for a second, mid C-terminal, SV binding site.

The finding that one of the SV binding sites is located in an alternatively spliced region, E44, raises the possibility that the two forms of CaV2.1 channels may have different functions with respect to transmitter release. While positive staining with PC2var antibody (*data not shown*), which is directed against the E44 sequence, argues that the splice-positive form is present at presynaptic terminals the question whether the other form, which we can presume exhibits weaker SV binding, is also presynaptic must be addressed in a future study.

The biochemical evidence presented here and elsewhere (Gardezi et al. in preparation) suggests both the CaV2.1 and CaV2.2 channel types, the two primary presynaptic calcium channels at fast transmitting synapses, exhibit C2 region SV binding. Further work will be necessary to determine the function of the both the C3 and C2 region SV binding sites. It would seem most likely, as suggested by Lübbert et al. (2017), that this site is important in the final stages of the SV recycling pathway but to what extent it participates in the alignment of the SV sensor with the channel to prime the release site for single-domain transmitter release gating will require further analysis.

## Ethics Statement

The research was exempt from ethics review as only unviable chicken embryos were used in this study.

## Author Contributions

CAS carried out almost all of the biochemical analyses and analyzed this data and prepared figures; contributed to the writing of the manuscript and academics. QL created the fusion protein constructs; cloned the chick CaV2.1 channel. BBE contributed to the cloning of the chick CaV2.1 channel, carried out informatics. HK-HM carried out immunocytochemistry. RHCC devised immunocytochemistry method for synaptosomes, contributed to immunocytochemistry, developed antibody, contributed to the writing of the manuscript and academics. SRG provided expertise on biochemistry and analysis, contributed to the writing of the manuscript and academics. EFS originated, supervised and critiqued project, carried out informatic analyses, wrote the manuscript and obtained the research funds.

## Conflict of Interest Statement

The authors declare that the research was conducted in the absence of any commercial or financial relationships that could be construed as a potential conflict of interest.
