# Supplementary material for: Molecular Characterization of an SV Capture Site in the Mid-Region of the Presynaptic CaV2.1 Calcium Channel C-Terminal
Source: Front Cell Neurosci. 2018 May 11;12:127. doi: 10.3389/fncel.2018.00127 (PMC5958201; doi:10.3389/fncel.2018.00127)
Supplement: Supplementary file 1 [file Data_Sheet_1.pdf]

## Supplemental Methods

***Fluorescent Immunostaining.***

*Chick cerebellar tissue slice preparation*. Fluorescent immunohistochemistry was carried out on E19 chick cerebellar slices. Cerebella were dissected and fixed in 2% paraformaldehyde and a series of 10% (4 h), 20% (1 h), and 30% (overnight) sucrose in PBS (1x phosphate buffered saline pH 7.4; ThermoFisher). The cerebella were then embedded in M1 Freezing Medium (ThermoFisher) and sectioned into 12 µm thick slices using a Leica CM3050 S cryostat.

*Chick cerebellar immunohistochemistry.* Tissue was washed 3x 10 min with PBS prior to being permeabilized in 0.1% Triton X-100 (Sigma-Aldrich, St.Louis, MO, USA) in PBS for 10 min. The cultures were washed with PBS three times and were blocked with 5% donkey serum in PBS for 50 min before antibody application. Primary antibodies were diluted in PBS before application as follows: PmidC2 1:200; PC2var, 1:200; SV2 monoclonal monoclonal in ascites fluid, 1:2; and calbindin monoclonal, 1:200. All secondary antibodies (Jackson ImmunoResearch, West Grove, PA) were used at a 1:100 dilution in PBS. Incubation for primary antibody was overnight at 4°C while incubation of secondary was at room temperature for 1 hr.

## Supplemental Figure 1A

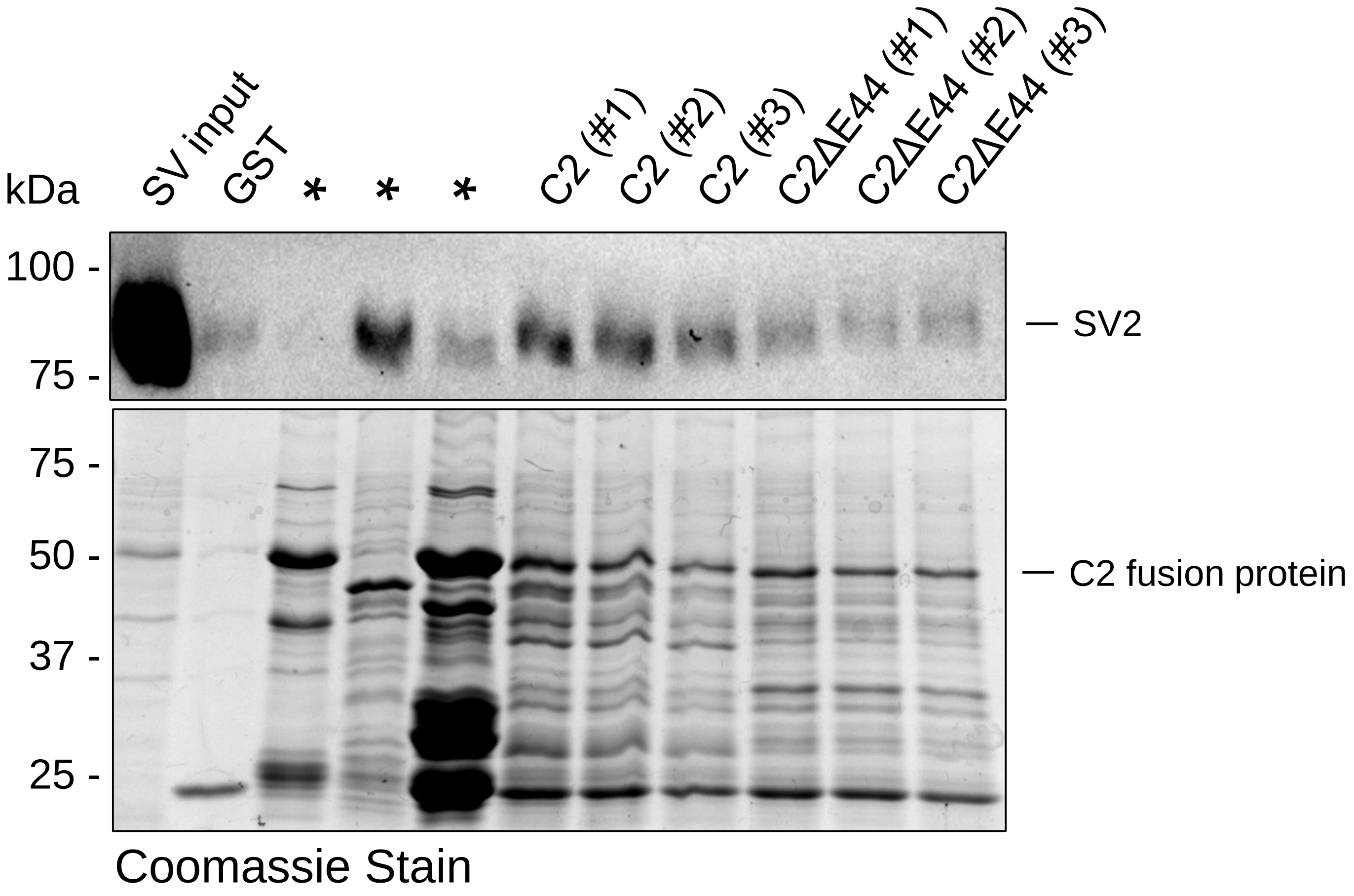


Representative example of a single SV-PD experiment immunoblot showing SV2 bands (upper panel) and a corresponding Coomassie stain of the fusion proteins (lower panel). Lanes marked '*' are from an unrelated experiment. In this case, we ran three different fusion protein concentrations for each test fusion protein, C2 and C2ΔE44 (as numbered).

**Supplemental Figure 1B (continued)**

**Original blots for SV-PD figures.** Lanes marked with an * are for unrelated experiments.

***C1-C2 SV-PD (Figure 4a)***

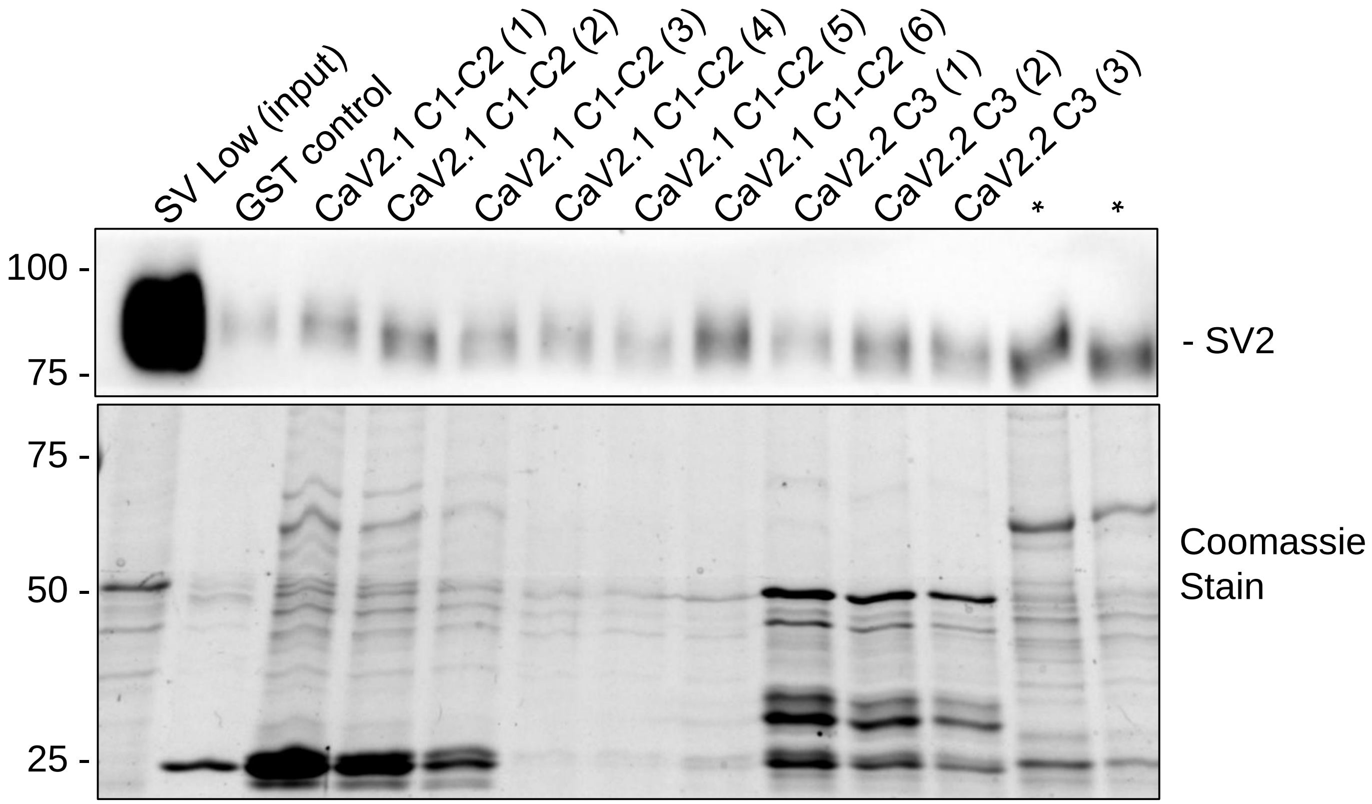


***C1 vs C2 SV-PD (Figure 4b)***

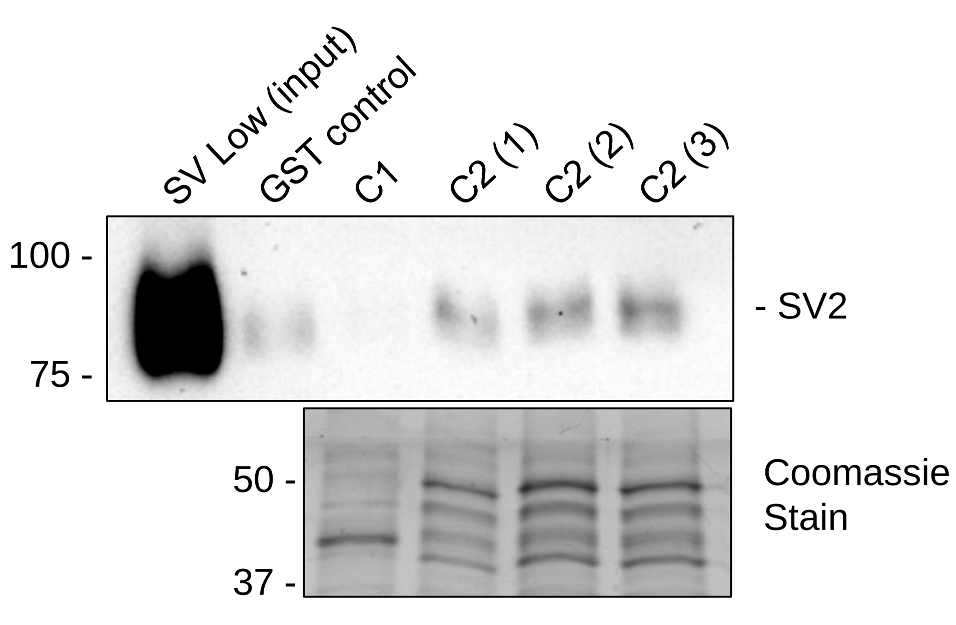

## Supplemental Figure 1B (continued)

***C2 and C2ΔE44 with peptides (Figures 5 and 7A)***

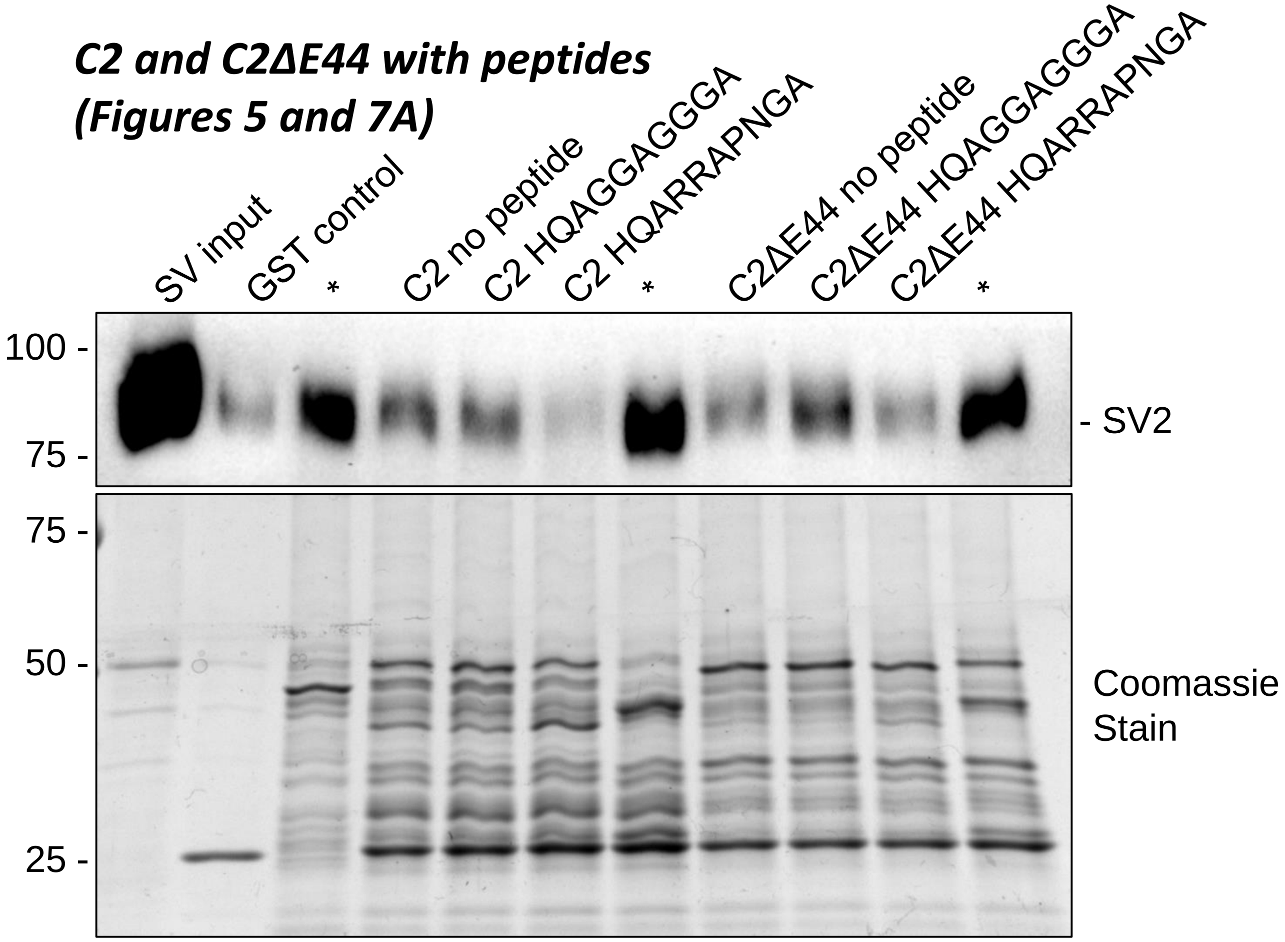


***C2 vs C2ΔE44 SV-PD (Figure 6A)***

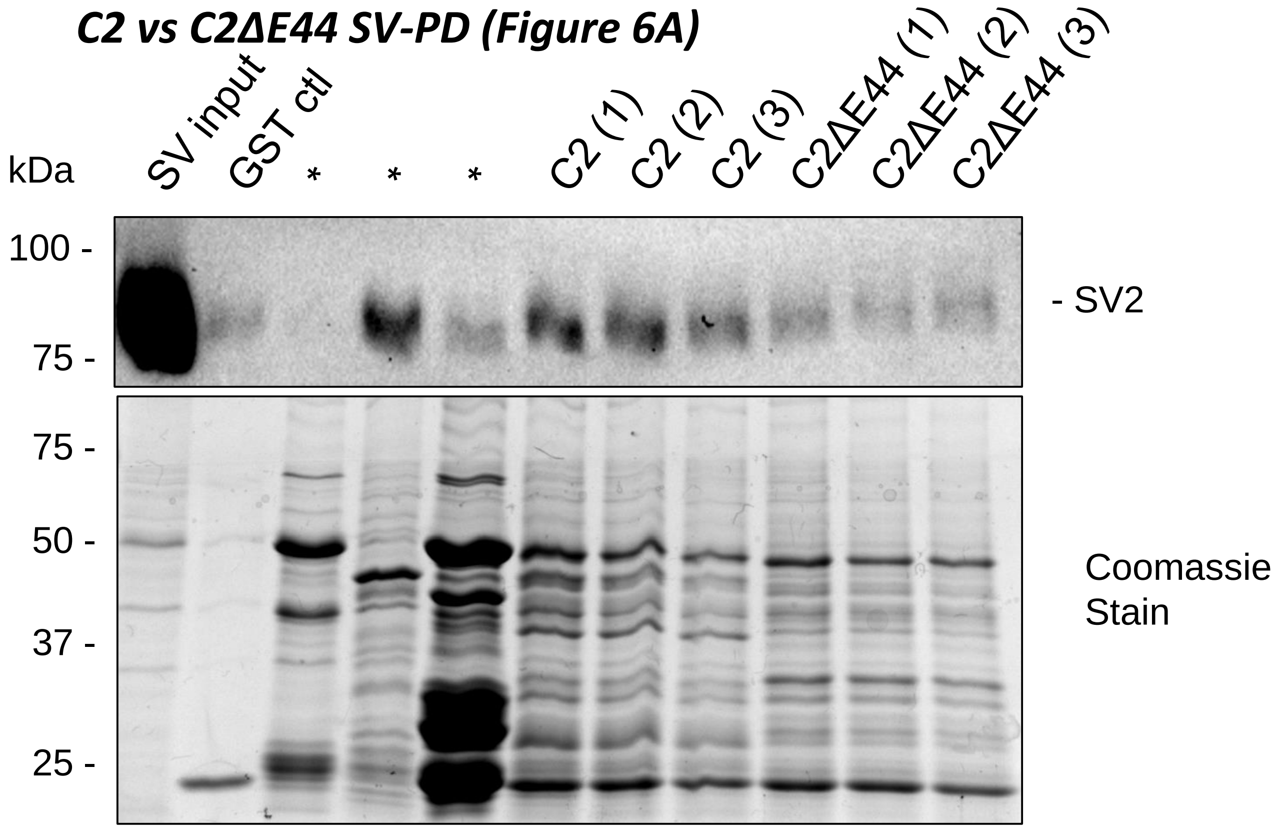

**Supplemental Figure 1B (continued)**

**C2 vs C2ΔE44 vs C2ΔE44Δ58-68 SV-PD and C2ΔE44Δ58-68 with peptides (Figures 6B and 7B)**

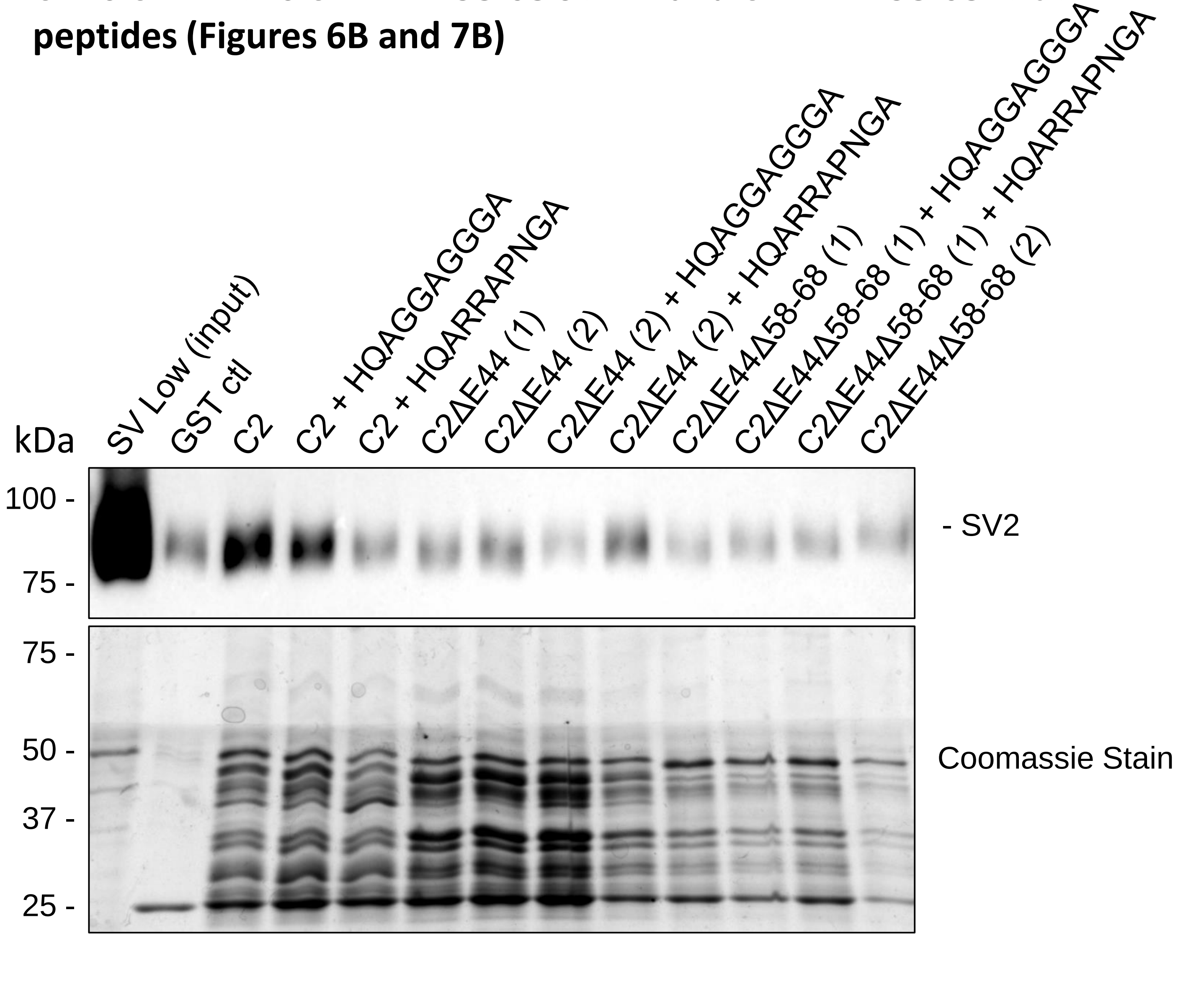

# Supplemental Figure 2

Gallus gallus CaV2.1 alpha 1 subunit.  The C-terminal is highlighted.
NCBI Reference Sequence: KY353011

```
/translation="MARFGDDHPSRYGAGGGGALGSSMGRVSGGSRAAGGGPGGGGGG
GAPPGGQRVYKQSMAQRARTMALYNPIPVRQNCLTVNRSLFLFSEDNAVRKYAKRITE
WPPFEYMILATIIANCIVLALEQHLPDDDKTPMSERLDDTEPYFIGIFCFEAGIKIIA
LGFAFHKGSYLRNGWNVMDFVVVLTGILATVGSQFDLRTLRAVRVLRPLKLVSGIPSL
QVVLKSIMKAMIPLLQIGLLLFFAILIFAIIGLEFYMGKFHTTCFDLVTNEIKVEVPC
GTDEPARICPNGTKCRKYWEGPNYGITQFDNILFAVLTVFQCITMEGWTDLLYYSNDA
SGNTWNWLYFIPLIIIGSFFMLNLVLGVLSGEFAKERERVENRRAFLKLRRQQQIERE
LNGYMEWISKAEEVILAEDEGEGEPRHPFDALRRATIKKSKTDLLSPEDAEEQLADIA
SVGSPFARASLKSAKLENATFFHKRERRMRFYIRRVVKTQAFYWTVLSLVALNTLCVA
IVHYDQPEWLSDFLYYAEFIFLGLFMSEMFIKMYGLGTRPYFHSSFNCFDCAVIIGSI
FEVIWAVVKPGTSFGISVLRALRLLRIFKVTKYWASLRNLVVSLLNSMKSIISLLFLL
FLFIVVFALLGMQLFGGQFNFDTGTPPTNFDTFPAAIMTVFQILTGEDWNAVMYDGIK
SQGGVKGGMVFSVYFIVLTLFGNYTLLNVFLAIAVDNLANAQELTKDEQEEEEAANQK
LALQKAKEVAEVSPLSAANLSIAVKEQQKNQKGSRSVWEQRTSELRKQNLLASREALY
GELEPEERWKPPYGRHLRPDAKTHRDRPLVVDPRENRNNNTNKTRPAVAADGAALRSA
LRRRAAPAAPPRRPRRPPPRRGARGRPAAPEPGGGARRRGRGAGEGAEAPRPGGARRV
AANGAPEAPRAPPGGGGRRGAALAAPRRGARGRGDGRRRALRLREAEAAPARSGGRRG
LRRRRPPGRQGEEAPQEAGEPRPRGAARAVHHAPHPAGPGPAAAARGGGHRQHEIPAC
PPRPPAPLKTIKRLPKLNGQPEGPPPPDDGLVVTNPTAHNDPTAALRRAEPKAEPKAE
PKAEHTAVEIPPLLPPPPSSALVQMNRNANPEPLPRKEEEEKKEEEGNGDEENGPKPM
VPYSSMFILSPTNPFRRLCHYIVNLRYFEMCILMVIAMSSIALAAEDPVQPNAPRNNV
LRYFDYVFTGVFTFEMVIKMVDLGLVLHQGAYFRDLWNILDFIVVSGALVAFAFTGSS
KGKDINTIKSLRVLRVLRPLKTIKRLPKLKAVFDCVVNSLKNVLNILIVYMLFMFIFA
VVAVQLFKGKFFYCTDESKEFEKDCRGEYLVYEKNEVKAQRREWKKYDFHYDNVLWAL
LTLFTVSTGEGWPQVLKHSVDATYENQGPSPGYRMEMSIFYVVYFVVFPFFFVNIFVA
LIIITFQEQGDKMMEEYSLEKNERACIDFAISAKPLTRHMPQNRQSFQYRMWQFVVSP
PFEYTIMAMIALNTIVLMMKFYDASDAYENVLKMFNNVFTSLFSLECLLKIMAFGVLN
YFRDAWNVFDFVTVLGSITDILVTEFGNNFINLSFLRLFRAARLIKLLRQGYTIRILL
WTFVQSFKALPYVCLLIAMLFFIYAIIGMQVFGNIGIEEEDDESAITQHNNFRTFFQA
LMLLFRSATGEAWHEIMLSCLSGKPCDENSGIKEDECGNEFAYFYFVSFIFLCSFLML
NLFVAVIRDSSILGPHHLDEYVRVWAEYDPAAWGRLTLMDMYAMLRNMSPPLGLGEKC
PPRVAYKRLLRMDLPVADDNTVHFNSTLMALIRTALDIKIAKGGADKQQMDAELRKEM
VAIWPNLSPKNLDLLVTPHKSTDLTVGKIYAAMMIMEYYRQSKAKKLQAMREEQNRTP
LMFQRMEPPSPTQEGPPGTDAAPTAEPAVRDGGIKESPSWVTQRAQEMFQRTGTWSPE
RGHEDVPNSRPNSQSVELREMPRDGSDGEYLPVEGHGRAASMPRLPADNQRRKVRPRG
NNLSTIADSPIRRSASTLGSGRGRAVRLDEFSLERIAPDGGQRHHPRRGHRGHRSSER
SGGRYTDGDTGLGTDLSITTQSGELPPPPPKDRDPERGRPKDRRHRHCMNRNN"
BASE COUNT      1280 a   2081 c   1921 g   1101 t
```

# Supplemental Figure 3

## A. PmidC2 antibody

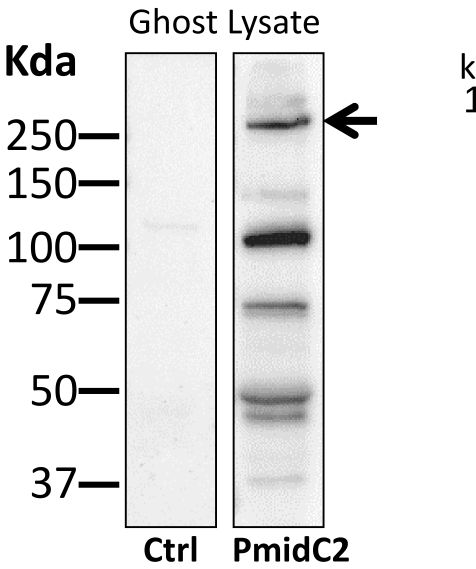


*i*. WBs of chick synaptosomes ghost (surface) membrane lysate probed with pre-immune or PmidC2 serum. Arrow indicates a band corresponding to the molecular weight of CaV2.1. Lanes are from the same blot.

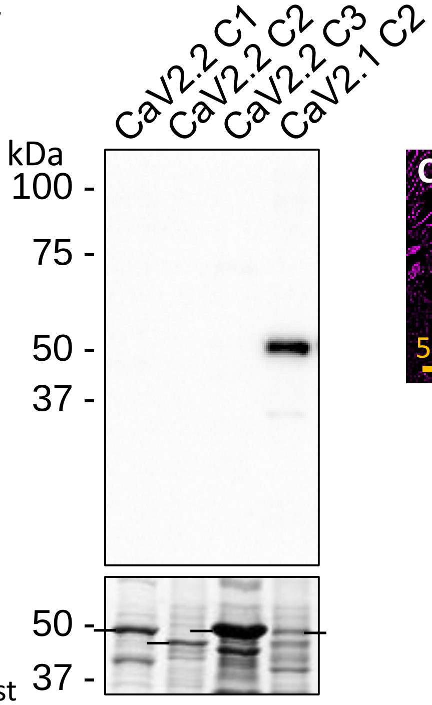


*ii*. Top panel: WBs of CaV2.2 C1, C2 or C3 and CaV2.1 C2 fusion proteins probed with PmidC2. Bottom panel: Coomassie stain of fusion proteins (horizontal black lines).

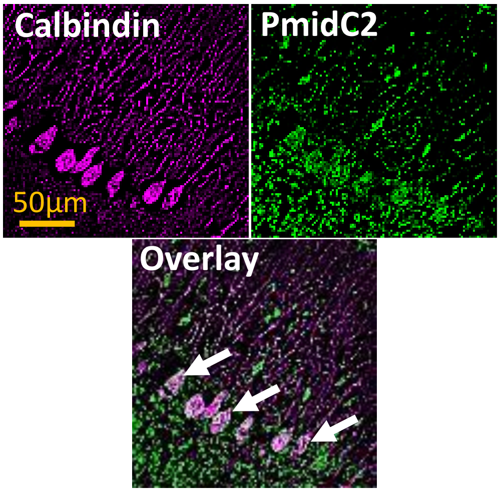


*iii*. Immunocytochemistry of chick cerebellar slices (10 µm) showing Purkinje neurons identified by calbindin and counter stained with PmidC2. Representative co-stained Purkinji neurons are indicated by white arrows.

## B. PC2var antibody

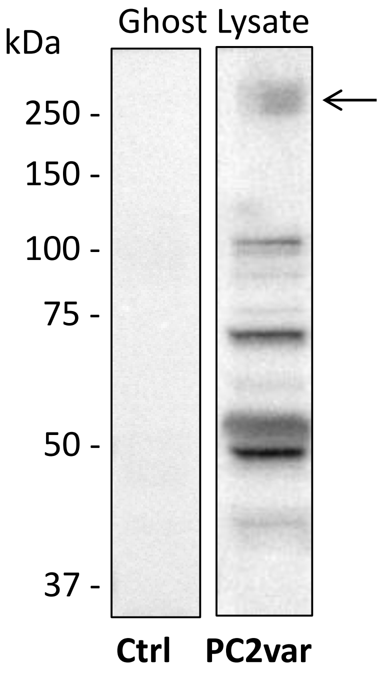


*i*. As in ***Ai*** But probed with PC2var.

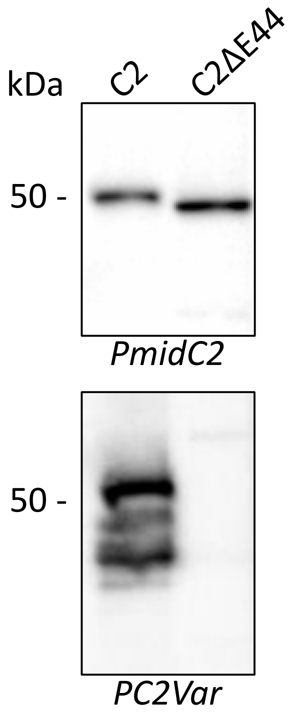


*ii*. WBs of full length, C2, and C2ΔE44 mutant fusion proteins probed with PmidC2 or PC2var, as labeled. PC2var does not recognize the splice variant.

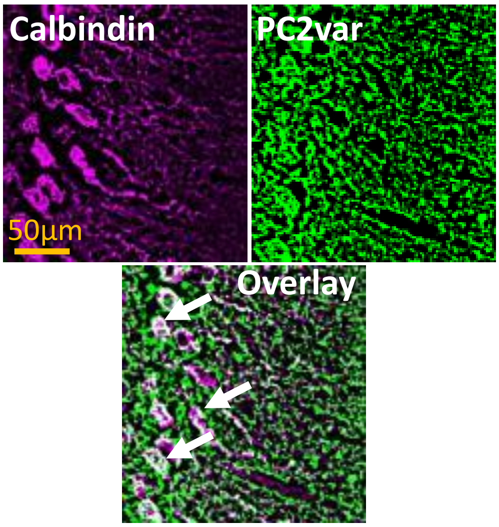


*iii*. As in ***Aii*** but counterstained with PC2var.

# Supplemental Table 1

**Forward and reverse primers used for the generation of CaV2.1 fusion proteins.**

| Construct | Primer Pair | Sequences (nucleotide) | Annealing Temperature (ºC) |
|---|---|---|---|
| **PCTERM** | PCtermF<br>PCtermR | GTGGTGGTGGAATTCGCGTTTGGGCCGAATACG<br>AGTAAGCTTGAGCTCATTATTATTTCGGTTCATGCAATGGC | 48 |
| **PCPROX** | PCproxF3<br>PCproxR | GTGGTGGTGGAATTCACCTGGATGAATACGTCCGCGTTTGG<br>AGTAAGCTTGAGCTCGGTTCTGCTCCTCCCGCATGGC | 58 |
| **PCMID** | PCmidF<br>PCmidR | GTGGTGGTGGAATT CGCACACCGCTGATGTTCCAACGC<br>AGTAAGCTTGAGCTCGGCAGCTCCCCCGATTGG | 60 |
